# Author Correction: Intradermal needle-free injection prevents African Swine Fever transmission, while intramuscular needle injection does not

**DOI:** 10.1038/s41598-023-34898-y

**Published:** 2023-05-15

**Authors:** Muhammad Salman, Hongyao Lin, Roypim Suntisukwattana, Parin Watcharavongtip, Patumporn Jermsutjarit, Angkana Tantituvanont, Dachrit Nilubol

**Affiliations:** 1 Swine Viral Evolution and Vaccine Development Research Unit, Department of Veterinary Microbiology, Faculty of Veterinary Science, Chulalongkorn University, Henry Dunant Road, Pathumwan, Bangkok, 10330 Thailand; 2 MSD Animal Health Innovation Pte Ltd, Singapore, 718847 Singapore; 3 Department of Pharmaceutic and Industrial Pharmacies, Faculty of Pharmaceutical Sciences, Chulalongkorn University, Bangkok, 10330 Thailand

Correction to: *Scientific Reports,* 10.1038/s41598-023-31199-2, published online 21 March 2023

The original version of this Article contained errors in the Material and methods section, under the subheading ‘Infectious inoculums’, where

“The ASF genotype II virus used in this study originated from infected pigs during a field outbreak in Thailand in 2021.”

now reads:

“The inoculum used in the study originated from infected pigs during a field outbreak in Thailand in 2022.”

Furthermore, under the subheading ‘Study design’ of the same section, the following sentences have been removed:

“All animal procedures were performed in compliance with the recommendations of the Guide for the Care and Use Laboratory Animal of the National Research Council of Thailand, according to the protocol reviewed and approved by the Chulalongkorn University Animal Care and Use Committee under protocol number 2031015. All methods were performed in accordance with the relevant guidelines and regulations.”

Lastly, the Acknowledgements section contained an error due to an incorrect grant fund number.

“This Research is funded by Thailand Science research and Innovation Fund Chulalongkorn University (FOOD66310018), Chulalongkorn University for Research Unit; Swine Viral Evolution and Vaccine Development Research Unit (GRU 3310160014), and Second Century Fund (C2F) for postdoc fellowship from Chulalongkorn University, Thailand.”

now reads:

“This Research is funded by Chulalongkorn University for Research Unit; Swine Viral Evolution and Vaccine Development Research Unit (GRU 3310160014), and Second Century Fund (C2F) for postdoc fellowship from Chulalongkorn University, Thailand. The authors are thankful to a technical assistance in a satellite laboratory.”

The original Article has been corrected.

